# Growth hormone in the tumor microenvironment

**DOI:** 10.20945/2359-3997000000186

**Published:** 2019-11-01

**Authors:** Vera Chesnokova, Shlomo Melmed

**Affiliations:** 1 Pituitary Center Department of Medicine Cedars-Sinai Medical Center Los Angeles CA USA Pituitary Center, Department of Medicine, Cedars-Sinai Medical Center, Los Angeles, CA, USA

**Keywords:** Growth hormone, tumor microenvironment, DNA damage, neoplastic cell transformation

## Abstract

Tumor development is a multistep process whereby local mechanisms enable somatic mutations during preneoplastic stages. Once a tumor develops, it becomes a complex organ composed of multiple cell types. Interactions between malignant and non-transformed cells and tissues create a tumor microenvironment (TME) comprising epithelial cancer cells, cancer stem cells, non-tumorous cells, stromal cells, immune-inflammatory cells, blood and lymphatic vascular network, and extracellular matrix. We review reports and present a hypothesis that postulates the involvement of growth hormone (GH) in field cancerization. We discuss GH contribution to TME, promoting epithelial-to-mesenchymal transition, accumulation of unrepaired DNA damage, tumor vascularity, and resistance to therapy. Arch Endocrinol Metab. 2019;63(6):568-75

## INTRODUCTION

Research into neoplastic formation is largely focused on genetic mutations resulting in benign or malignant tumors. However, neoplastic transformation begins long before the cancer lesion is detected. Most mutations occur in the preneoplastic stage of cancer development (1) and local mechanisms allowing for these somatic mutations are poorly understood. Tumor-independent processes may alter the local milieu and help create a microenvironment sufficiently receptive to develop precancerous and cancerous changes. This process, termed “field cancerization”, was introduced by Slaughter and cols. (2) in reference to replacement of normal epithelial cell populations with cancer-primed populations, but later defined as “a somatic evolutionary process that produces cells that are closer to cancer” (3). With chronic inflammation, for example, colonic mucosa of patients with inflammatory bowel disease undergoes a “field change”, creating a favorable environment for genetic mutations before histological dysplasia is evident (4-6).

Developed tumors are composed of multiple cell types that interact with one another. Interactions between malignant and non-transformed cells and tissues create a tumor microenvironment (TME) (7) comprising epithelial cancer cells, cancer stem cells, non-tumorous cells, stromal cells (including resident fibroblasts, cancer-associated fibroblasts, adipose cells, and pericytes), immune-inflammatory cells, blood and lymphatic vascular network, and extracellular matrix (ECM) (7-10). TME was consistently shown to play a role in evolution of malignancies, and tumor development is highly dependent on the specific TME. Rapid expansion of tumor cells triggers hypoxia, resulting in metabolic reprogramming of tumor cells; interplay between cancer and neighboring cells results in further alteration of TME cellular components, restructuring of ECM, and formation of disorganized vascularization systems. Cancer TME constituents adapt to environmental conditions, promoting overall tumor growth (9,11).

Multiple factors shape the environment to enable field cancerization in normal tissue or for tumor cell evolution toward malignization in TME. Growth hormone (GH) is secreted from pituitary somatotrophs and can also be expressed in non-pituitary tissue. Many GH actions are mediated by the insulin-like growth factor (IGF)/IGF receptor (IGFR) pathway, although GH exerts IGF-independent effects in bone, muscle, liver, and colon tissues (12-14). GH may create a protumorigenic environment in normal epithelial cells, suppressing tumor suppressor proteins and promoting neoplastic transformation (15,16). In neoplastic tissue, local GH expression has been linked to several malignancies and several excellent reviews describe endocrine and autocrine/paracrine tumor-promoting GH actions in cancer cells and tissues (17-20). In this brief review, we focus on mechanisms underlying pro-oncogenic actions of GH as a field modifier in non-transformed cells and as a tumor promoter in TME.

## GH AND FIELD CANCERIZATION

Field cancerization occurs in response to exogenous or endogenous insults, mutagen exposure, or age-related mutations in non-transformed cells. Although changes such as increased growth rate and decreased death rate may occur, cells do not display dysplasia (3). With further genetic alterations, preneoplastic cells evade normal growth-control mechanisms and clonal selection ultimately leads to development of a malignant clone (1,21). We suggest a broader definition of field cancerization, which includes cells with “phenotypic alterations required for malignancy” (3), as well as the process and mechanisms by which these yet undetectable early changes occur.

DNA damage response (DDR) and DNA repair protect cells from chromosomal instability and ultimately cancer. DDR signaling pathways react to endogenous or exogenous DNA damage and coordinate complex DNA repair processes (10). Thus, DDR genes are considered “caretakers” of the genome, as most oncogenic alterations are caused by inadequate DNA repair (22) and acquisition of oncogenic mutations with sustained proliferation. DNA damage also accumulates with age due to attenuated DNA repair mechanisms (23).

Initiation of DDR starts upon recruitment of the MRE11/RAD50/NSB1 protein complex to the site of DNA damage, which, in turn, activates phosphoinositide-3-kinase-related kinases: ataxia-telengiectasia mutated (ATM), ATM and Rad3 related (ATR), and DNA-dependent protein kinase (DNA-PK) (24,25). These kinases phosphorylate and activate proteins essential for DNA repair, including H2AX, BRCA1, BRCA2, and TERT (26-28). ATM and ATR also phosphorylate checkpoint kinases Chk2 and Chk1, arresting cell proliferation, as well as the tumor suppressor p53 facilitating DNA repair, apoptosis, or cell cycle arrest (29,30).

In non-pituitary cells, GH expression is very low, but can be significantly induced and secreted in response to DNA damage pathway activation (31). In non-tumorous human colon and mammary cells, murine colon tissue, and 3-dimensional human intestinal organoids derived from induced pluripotent stem cells, GH attenuates DDR, decreasing ATM kinase activity as well as Chk2 and p53 phosphorylation, which subsequently reduces DNA repair by both homologous recombination (HR) and non homologous end joining (NHEJ), resulting in accumulated unrepaired DNA (16). Non-tumorous human colon cells exposed to GH generate more colonies in soft agar, an indication of cell transformation, and mice bearing xenografts secreting GH develop more metastases (16). Peripheral blood lymphocytes of acromegaly patients harboring GH-secreting pituitary adenomas also exhibit increased chromosomal aberrations (32,33), and unrepaired DNA damage accumulates in the liver in a zebrafish model of acromegaly. Thus, in normal cells and tissues, elevated GH, whether secreted or induced locally, suppresses DNA repair, enabling an environment favorable for accumulation of oncogenic mutations and chromosomal instability.

If DNA damage repair is not optimal, cells continue to proliferate, usually acquiring oncogenic mutations, undergo apoptosis, or exit the cell cycle and become senescent, thereby limiting propagation of damaged cells (34). Senescent cells remain metabolically active, secreting cytokines, chemokines, matrix metalloproteinases (MMPs), IGF1, IGF8, IGF binding proteins, and other factors as part of the senescence-associated secretory phenotype (SASP) (35,36). Senescent cells also increase with age-associated attenuation of DNA damage repair (37-39). Secretion of SASP may persist, affecting neighboring cells (40-42). We showed that GH is also a component of SASP (31), and GH secretion from senescent cells alters DDR activity in surrounding tissues, favoring DNA damage accumulation as evidenced by increased levels of DNA damage observed in senescent cells (43).

Another possible role for GH in field cancerization lies in its ability to suppress tumor suppressor proteins. GH results in decreased expression of p53, PTEN, and APC in human non-tumorous colon cells and 3-dimensional human intestinal organoids, while suppressing GH signaling with the GH receptor (GHR) antagonist pegvisomant led to p53 induction in colon tissue of acromegaly patients (15). Similarly, crossbreeding of *Apc*^*+/-*^ mice, which all develop multiple intestinal and colon tumors by 9 months of age, with *Ghr*^*-/-*^ mice markedly decreased the number and size of tumors due to elevated colon and intestinal p53 expression in the double mutant *Apc*^*+/-*^*Ghr*^*-/-*^ mice (15). GH-induced decreased PTEN may trigger mTOR activity (44), stimulating cell proliferation and survival, while p53 deficiency may also enhance proliferation, exacerbating GH effects on DNA damage accumulation.

In summary, excess GH secreted from somatotroph pituitary adenomas or GH induced locally in response to DNA damage, senescence, or inflammation (15) may alter the local microenvironment, providing a favorable milieu for non-transformed cells to acquire pro-proliferative mutations.

## GH ACTIONS AS A PART OF TME

### GH and Epithelial-Mesenchymal Transition (EMT) in TME

EMT, a developmental regulatory program triggered in cancer cells that results in epithelial cell transformation, enables cells to acquire the ability to invade, resist apoptosis, and proliferate (10,45). Pleiotropically acting transcription factors including Snail, Slug, Twist, and Zeb1/2 orchestrate EMT, suppressing expression of E cadherin, which is involved in cell-to-cell adhesion, and inducing the mesenchymal marker N cadherin, thereby promoting motility and invasiveness (10,46). Secreted MMPs, a multigene family of zinc-dependent ECM remodeling endopeptidases, are also implicated in the multistep processes of invasion and metastasis, with ECM degradation, migration, and angiogenesis promoting tumor progression (47).

The role of GH in EMT was analyzed in depth in a recent review (20). Here, we briefly recount studies on the effects of GH on EMT in several cancer models. GH transcription and protein expression was documented in human breast cancer and endometrial tissue (19,48,49) and in hepatocellular carcinoma (50), while GHR is expressed in several human cancers (51). Enhanced expression of GH releasing hormone and its receptor was found in cancer cell lines and in human malignant tissue (52,53). In human hepatocarcinoma cells, elevated GH promotes cell migration and invasion by inhibiting transcription of Claudin1, a tight junction component (54). In human breast cancer MCF7 cells, autocrine/paracrine GH promotes MMP2 and MMP9 metalloprotease release and an EMT phenotype (55). Forced expression of GH induces TFF3, which in turn, enhances anchorage-independent growth, a marker of cell transformation (56). In these cells, GH overexpression was not associated with induced IGF-1 (57), suggesting a direct effect of GH in EMT. Further, both GH and WNT4 are upregulated in human mammary carcinoma and tumor xenografts expressing GH. Autocrine GH stimulates WNT4 expression in breast cancer cells, which, in turn, increases mesenchymal markers vimentin, MMP2, and MMP7, while inducing cell migration and suppressing apoptosis (58). Finally, the microRNA 96-182 cluster, which promotes EMT and invasion by directly suppressing breast cancer metastatic suppressor 1-like gene expression via STAT3 and STAT5 signaling, is enhanced in human metastatic breast cancer (59), and microarray profiling in breast cancer cells shows that autocrine GH induces this microRNA cluster.

GH and GHR are abundantly expressed in human melanoma cells, and treatment with GH resulted in decreased E cadherin and increased N cadherin, while GHR knockdown reversed the effect (60). Conversely, silencing GH signaling in human pancreatic ductal adenocarcinoma cell lines resulted in increased E cadherin, while EMT markers including N cadherin, Zeb, Snail, and Slug were suppressed (61).

Nuclear localization of GHR and increased GHR levels have been reported in breast and colorectal carcinoma (62,63). In Ba/F3 murine lymphocyte cells, nuclear GHR localization was associated with oncogenic transformation and tumor metastasis due to enhanced nuclear translocation of phosphoSTAT5 generated at the cell surface by autocrine GH (64). In human colorectal cancer tissue, GH expression was associated with metastases, and forced GH expression with increased transcription of fibronectin 1, a mesenchymal marker, as well as decreased expression of E cadherin, followed by increased migration and invasion (65). GH was not induced in human colon adenocarcinoma tissue, but GHR was significantly upregulated in cancer cells compared to normal adjacent colon tissue (15). Nevertheless, in human HCT116 colon adenocarcinoma cell as well as in non-tumorous colon cells, GH treatment promoted induction of EMT transcription factors Snail and Twist2, respectively, while decreasing E cadherin, cell migration, and invasion. Importantly, co-culturing human colon adenocarcinoma HCT116 cells with GH-expressing human colon fibroblasts resulted in increased HCT116 cell migration and soft agar colony formation (15). Thus, elevated GH, a paracrine component of TME, may initiate or exacerbate EMT, enhancing metastatic potential.

### GH and DNA damage in TME

Multiple mutations in malignant tumor DNA repair pathways are associated with DNA damage (66). GH induced in human breast carcinoma MDA-MB-436S and T47D cells as well as endometrial carcinoma RL95-2 cells increases clonogenicity and attenuates radiation-induced or mitomycin-induced DNA damage by activating DNA damage repair genes *BRCA1*, *BRCA2*, and *TERT*, promoting tumor cell survival. Accordingly, malignant cell GH induction in response to DNA damage may contribute to TME, resulting in tumor chemotherapy or radiation resistance (67,68).

By contrast, suppressing GH signaling attenuates DNA repair, allowing DNA damage to accumulate. These cells are more prone to apoptosis and thus more sensitive to DNA damaging therapy (67,68). Accordingly, GH induction in response to DNA damage may contribute to TME, resulting in tumor chemotherapy or radiation resistance.

### GH and tumor vascularization in TME

Tumor vascularization is an important part of TME, and angiogenesis promotes tumor progression, invasion, and metastasis (69). Recent studies suggest that tumor cells secrete soluble factors that attract blood vessels to increase blood supply and enhance metastasis (70). In benign and malignant vascular tumors, including angiosarcoma, Kaposi’s sarcoma, hemangioendothelioma, and hemangioma, GHR is significantly upregulated in both cytoplasm and nuclei, implying that tumor cells are targets for GH action. Indeed, GH exhibits mitogenic effects in vascular tissue cells, including smooth muscle cells, fibroblasts, and endothelial cells (71). In human mammary carcinoma MCF7 cells, GH promoted VEGF-A expression via an autocrine/paracrine effect and subsequent *in vitro* tube formation in human microvascular endothelial cell line; *in vivo*, in a xenograft model of human mammary carcinoma, autocrine/paracrine GH increased tumor blood and lymphatic microvessel density (72). Although GH may act independently in TME, it has been shown in colorectal carcinoma to upregulate VEGF expression via IGF-1 induction (73,74).

### GH and immune cells in TME

Tumor cells secrete chemokines and cytokines into the microenvironment to recruit and activate immune cells. In turn, activated immune cells form a cancer-related inflammatory microenvironment promoting tumor progression (75). Macrophages comprise the majority of immune cells in this microenvironment (8). GH was shown to stimulate macrophage motility in several *in vitro* models (76) and serves as a chemoattractant for human monocytes (77) and T cells. Indeed, GH-secreting pituitary adenomas contain significantly more CD4+ and CD8+ T cells than do non-GH adenomas (78). Thus, GH, induced in tumor cells via a paracrine effect, may contribute to inflammatory aspects.

Crosstalk between cells in the neoplastic microenvironment may support cancer cell capability for invasive growth (79). In light of the evidence presented above, it is reasonable to conclude that GH expressed and secreted from tumor, stroma, or inflammatory cells likely plays a substantial role promoting EMT and transforming TME.

### GH, TME, AND RESISTANCE TO THERAPY

The goal of chemo- and radiation antitumor therapy is to cause cell death. However, TME may modulate responses to cytotoxic therapy, and GH, as a part of TME, may contribute to this process. GH effects on therapy resistance to cancer has been well described (80). Here, we elaborate on additional aspects of GH actions contributing to its effects on treatment resistance.

DNA damaging agents trigger DNA damage in tumor cells and also in neighboring non-tumorous epithelial or stromal cells, which likely results in GH upregulation. In non-tumorous epithelial cells, GH, acting in a paracrine/autocrine fashion, may suppress DNA damage repair, leading to DNA damage accumulation with potential oncogenic transformation. In fibroblasts, DNA damaging agents can trigger senescence, accompanied by SASP induction (81). GH, as a component of SASP, can attenuate effects of chemo- or radiotherapy by decreasing p53-dependent apoptosis in tumor cells (15). In MDA-MB-231 and MCF7 human mammary carcinoma cells, GH induced chemoresistance to doxorubicin by suppressing apoptosis, and these effects were reversed by the GHR antagonist pegvisomant (82,83).

GH may also impact chemotherapy resistance via its effects on multi-drug efflux pumps, which transport xenobiotics out of the cytoplasm (80). For example, GH expression in four different human melanoma cell lines upregulated expression of multiple ABC-family multi-drug efflux pumps, rendering cells resistant to chemotherapy (84).

### CONCLUDING REMARKS

**Figure 1:**
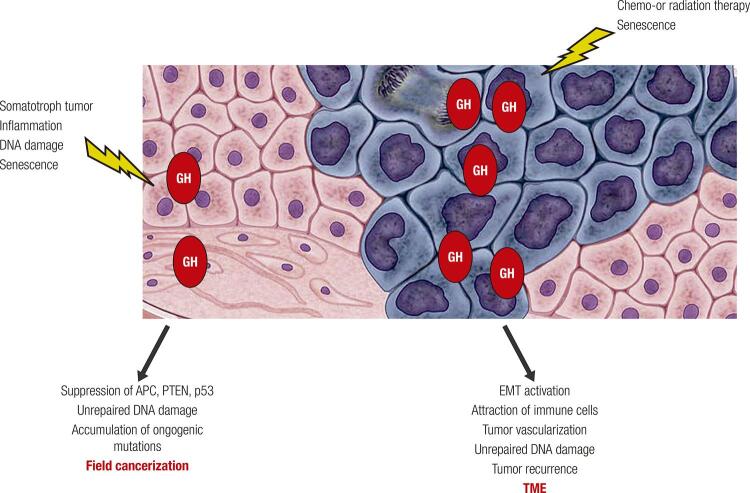
GH effects in non-tumorous tissue and TME. GH can be secreted by somatotroph pituitary adenoma cells, induced locally in non-tumorous tissue in response to DNA damage or inflammation, or secreted by senescent cells. By suppressing tumor suppressor proteins and altering DNA damage repair, GH promotes “field cancerization” in non-transformed cells, creating a pro-tumorigenic environment. Within the tumor, GH can be upregulated after DNA damaging therapy or in senescent cells, and, via autocrine/paracrine action, triggers tumor cell EMT, attracts immune cells, and promotes tumor vascularization, enabling survival, proliferation, and malignization of existing tumor cells. By enhancing unrepaired DNA damage in non-transformed neighboring cells, GH may promote tumor recurrence after treatment. Normal cells depicted in pink; tumor cells depicted in blue.

GH, either directly or by induction of IGF-1, promotes developmental growth, cell proliferation, differentiation, and survival (85-88). With age, activity of the somatotroph axis declines, which, from an evolutionary perspective, may be protective to safeguard the organism from potentially harmful effects of GH on age-related waning effectiveness of DNA repair pathways. Evidence presented here illustrate the emerging understanding of mechanisms implicating GH in promoting an environment favorable for neoplastic growth as well as in enabling proliferation and survival of existing tumor cells (Figure 1).

The proposed role for GH in field cancerization may explain the increased propensity of acromegaly patients to develop colon, skin, thyroid, and prostate tumors (89,90), as well as the appearance of changes consistent with hepatocellular carcinoma (91-93) and mammary adenocarcinoma (94) in transgenic animal models of GH excess. Furthermore, patients with inherited GH signaling deficiency (Laron syndrome) do not develop cancer (95), and GH- or GHR-deficient animal models live longer and are resistant to age-related or chemically induced tumors (12,96).

Microenvironmental factors, including GH, attenuate the efficacy of anticancer therapy. Effects of GH in TME have mostly been demonstrated *in vitro*, requiring further studies to confirm the role of GH in TME *in vivo*. Such studies will open new avenues for controlling the rate and direction of tumor cell evolution, and the potential for therapeutically targeting GH to improve anticancer therapy.
